# Laparoscopic management of a cavitated noncommunicating rudimentary uterine horn of a unicornuate uterus: a case report

**DOI:** 10.1186/1752-1947-4-215

**Published:** 2010-07-19

**Authors:** Ignacio Zapardiel, Pilar Alvarez, Tirso Perez-Medina, Jose M Bajo-Arenas

**Affiliations:** 1 Department of Obstetrics and Gynecology, Santa Cristina University Hospital, Madrid, Spain

## Abstract

**Introduction:**

A unicornuate uterus with a rudimentary horn is the most uncommon uterine anomaly of the female genital tract. It has an estimated frequency of one in 100,000 among the fertile female population. This anomaly results from the abnormal maturation of one Müllerian duct with the normal development of the contralateral one.

**Case presentation:**

We report here the case of a 14-year-old Caucasian girl who came to our hospital with intense dysmenorrhea. Imaging techniques revealed a unicornuate uterus with a rudimentary horn and a large hematosalpinx. We performed a laparoscopic removal of this uterine anomaly without any complication in the postoperative period.

**Conclusion:**

In our case report, we demonstrate that laparoscopy is the best approach for the treatment of IIb Müllerian abnormalities. Laparoscopy resulted in anatomical and reproductive results equivalent to those offered by a laparotomic approach, but with the additional advantages of minimally invasive surgery, such as better cosmetic results and postoperative period, which are essential for very young patients.

## Introduction

A unicornuate uterus with a rudimentary horn is the most uncommon uterine anomaly of the female genital tract. It has an estimated frequency of one in 100,000 among the fertile female population [1]. This anomaly results from the abnormal maturation of one Müllerian duct with the normal development of the contralateral one. There are few cases published and just one short case series [2]. We report the case of a laparoscopic removal of this uterine anomaly that was associated with a large hematosalpinx.

## Case presentation

**Figure 1:**
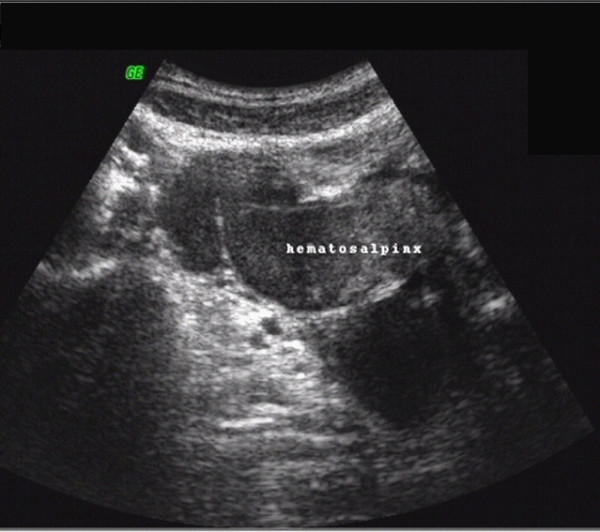
**Sonographic scan revealing a right hematosalpinx**. The image shows a 2 D vaginal ultrasound scan performed on the patient eight days before surgery. It reveals a hematosalpinx, measuring 45 × 33 mm, in the right Fallopian tube.

**Figure 2:**
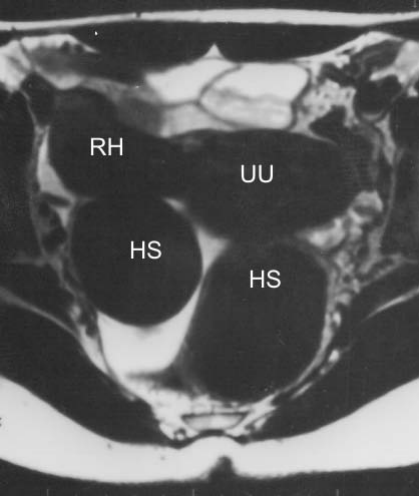
**Magnetic resonance imaging showing the unicornuate uterus (UU), the rudimentary horn (RH), and the looped hematosalpinx (HS)**. T1-weighted MRI of pelvis acquired six days before surgery. The MRI shows the uterus (UU) with a rudimentary horn (RH) in the upper side of the image. The dilated and looped Fallopian tube (HS) can be seen posteriorly to the UU and RH.

**Figure 3:**
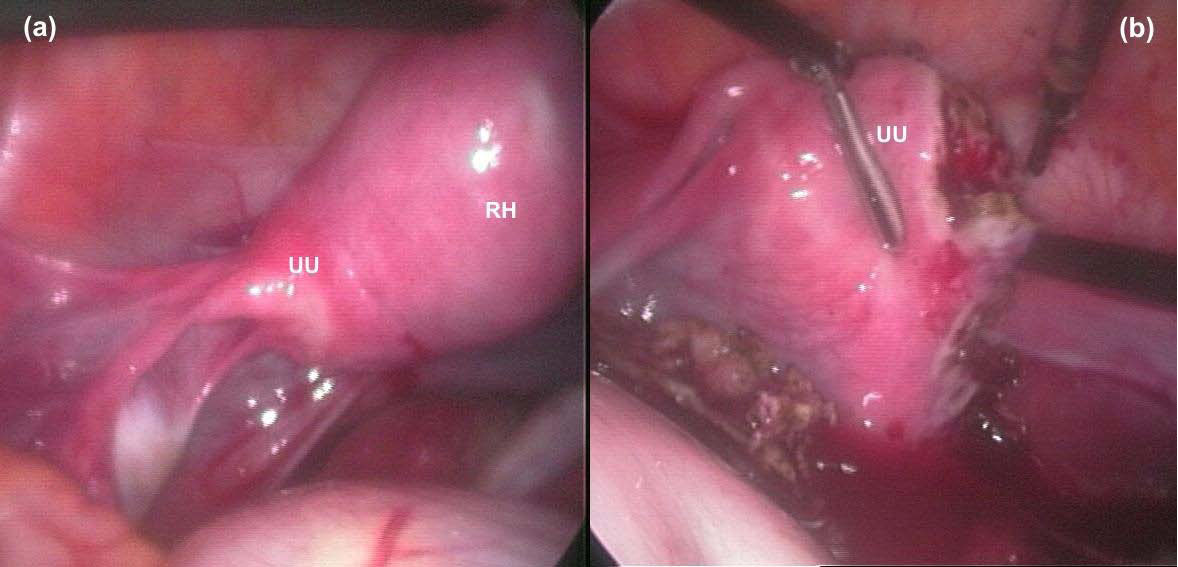
**Anatomy before (a) and after (b) removal of the rudimentary horn**. Unicornuate uterus (UU), rudimentary horn (RH). The pictures were taken during laparoscopic surgery. Before surgery (a) and after surgery (b) with the rudimentary horn removed.

A Caucasian 14-year-old girl was admitted to our department with a clinical history of dysmenorrhea for the past two years since her menarche. During her menstruation, she suffered from acute and severe right lower-quadrant pain that caused repeated school absenteeism. The rest of her medical history was unremarkable. Physical examination revealed a uterine mass in the right side of the pelvis with diffuse tenderness of the lower abdomen, but a normal vagina without masses and just one cervix. Three-dimensional ultrasonographic exam revealed the presence of a supposed didelphys uterus with hematometra in the right one, as well as a hematosalpinx measuring 45 × 33 mm on the same side (Figure 1). A magnetic resonance was carried out, showing a unicornuate uterus with a rudimentary horn, hematometra, and an ipsilateral hematosalpinx (Figure 2). No renal abnormalities were found. A laparoscopic procedure was performed, evidencing the expected imaging findings. Consequently, removal of the rudimentary horn and the right Fallopian tube (Figure 3) was carried out, while the right ovary and the entire left ovary and Fallopian tube were conserved. Pathologic reports of the surgical specimens did not show any abnormal tissue. The postoperative course was uneventful and the patient was discharged from the hospital in 48 hours. The patient remained asymptomatic in subsequent follow-ups.

## Discussion

Uterine Müllerian abnormalities occur when the embryo is just 20 mm long. All the anatomical variations are classified by the American Society of Reproductive Medicine. A unicornuate uterus with a noncommunicating rudimentary horn belongs to group II-b, which may be differentiated by a complete uterine septum bicollis with an obstructed longitudinal vaginal septum. This is diagnosed by a physical vaginal exploration as we did in this case. This subtype of anomaly is associated with endometriosis, by means of a retrograde menstruation, and also with renal abnormalities, both of which should be assessed before any treatment [3,4]. It is not clear whether a unicornuate uterus occurs more often on the right side. While some manuscripts report a frequency of 80% for the right-sided type, others report an estimated frequency of 62% [2]. In our present case, we observed a right-sided malformation with a firm attachment to the unicornuate uterus. In such cases, it is mandatory to perform a complete and strict preoperative evaluation and diagnosis. These patients usually present with diffuse pelvic pain and dysmenorrhea caused by hematometra in the cavitated rudimentary horn (such as the one described in our patient) endometriosis, pyometra, or torsion of the abnormal portion or the Fallopian tube. These patients also have a higher rate of ectopic pregnancies and miscarriage, as well as preterm deliveries when they succeed in getting pregnant [1]. With regards to imaging techniques, magnetic resonance imaging (MRI) seems to be the gold standard for the diagnosis of Müllerian abnormalities, although three-dimensional sonography is able to achieve comparable results [2,3,5]. We performed both techniques for the diagnosis of this patient. These techniques provided us with a detailed structure of the anatomical anomaly, allowing us to plan the surgical procedure in advance.

The surgical technique of choice has to be adapted to the type of malformation. In the case of a rudimentary uterine horn firmly joined to a unicornuate uterus, some details need to be kept in mind: First, there is not a well-defined limit between the unicornuate uterus and the rudimentary horn in some cases, which means the surgeon must proceed with extreme caution. Hysteroscopic transillumination can be useful in such a case, since opening the uterine cavity may affect future reproduction [1]. Second, the Fallopian tube on the side of the rudimentary horn must be removed in order to avoid tubal pregnancies [5]. Finally, it is important to consider that the ipsilateral ureter is closer to the uterine body, with a higher risk of lesion during rudimentary horn removal, and should be identified before the dissection [1]. Thus, we first removed the Fallopian tube with the hematosalpinx to have better access to the rudimentary horn. Subsequently, a careful dissection of the rudimentary horn was carried out with the precautions previously described. Finally, we sutured the myometrium in multiple layers and then the visceral peritoneum. No hysteroscopy was used in our patient.

## Conclusion

Our report demonstrates the superior performance of laparoscopy for the treatment of IIb Müllerian abnormalies. Laparoscopy resulted in anatomical and reproductive results equivalent to those offered by a laparotomic approach, but with the additional advantages of minimally invasive surgery, that is less scarring and a shorter postoperative period, which are essential for very young patients.

## Competing interests

The authors declare that they have no competing interests.

## Authors' contributions

IZ collected, analyzed, and interpreted data from the patient, and was a major contributor to writing the manuscript. PA and TP contributed to writing the manuscript and performed the surgery. JBA supervised the work and collected the patient's informed consent. All authors read and approved the final manuscript.

## Consent

Written informed consent was obtained from the patient's mother for publication of this case report and any accompanying images. A copy of the written consent is available for review by the Editor-in-Chief of this journal.
